# HD CAGnome: A Search Tool for Huntingtin CAG Repeat Length-Correlated Genes

**DOI:** 10.1371/journal.pone.0095556

**Published:** 2014-04-21

**Authors:** Ekaterina I. Galkina, Aram Shin, Kathryn R. Coser, Toshi Shioda, Isaac S. Kohane, Ihn Sik Seong, Vanessa C. Wheeler, James F. Gusella, Marcy E. MacDonald, Jong-Min Lee

**Affiliations:** 1 Center for Human Genetic Research, Massachusetts General Hospital, Boston, Massachusetts, United States of America; 2 Massachusetts General Hospital Cancer Center, Charlestown, Massachusetts, United States of America; 3 Children’s Hospital Informatics program, Children’s Hospital, Boston, Massachusetts, United States of America; 4 Center for Biomedical Informatics, Harvard Medical School, Boston, Massachusetts, United States of America; 5 i2b2 National center for Biomedical Computing, Boston, Massachusetts, United States of America; University of Florida, United States of America

## Abstract

**Background:**

The length of the huntingtin (*HTT*) CAG repeat is strongly correlated with both age at onset of Huntington’s disease (HD) symptoms and age at death of HD patients. Dichotomous analysis comparing HD to controls is widely used to study the effects of *HTT* CAG repeat expansion. However, a potentially more powerful approach is a continuous analysis strategy that takes advantage of all of the different CAG lengths, to capture effects that are expected to be critical to HD pathogenesis.

**Methodology/Principal Findings:**

We used continuous and dichotomous approaches to analyze microarray gene expression data from 107 human control and HD lymphoblastoid cell lines. Of all probes found to be significant in a continuous analysis by CAG length, only 21.4% were so identified by a dichotomous comparison of HD versus controls. Moreover, of probes significant by dichotomous analysis, only 33.2% were also significant in the continuous analysis. Simulations revealed that the dichotomous approach would require substantially more than 107 samples to either detect 80% of the CAG-length correlated changes revealed by continuous analysis or to reduce the rate of significant differences that are not CAG length-correlated to 20% (n = 133 or n = 206, respectively). Given the superior power of the continuous approach, we calculated the correlation structure between *HTT* CAG repeat lengths and gene expression levels and created a freely available searchable website, “HD CAGnome,” that allows users to examine continuous relationships between *HTT* CAG and expression levels of ∼20,000 human genes.

**Conclusions/Significance:**

Our results reveal limitations of dichotomous approaches compared to the power of continuous analysis to study a disease where human genotype-phenotype relationships strongly support a role for a continuum of CAG length-dependent changes. The compendium of *HTT* CAG length-gene expression level relationships found at the HD CAGnome now provides convenient routes for discovery of candidates influenced by the HD mutation.

## Introduction

Huntington’s disease (HD, OMIM # 143100) is an autosomal dominant neurodegenerative disorder caused by an expansion of a polymorphic CAG trinucleotide repeat in the first exon of huntingtin (*HTT*), the gene encoding huntingtin protein [1]. There is a strong inverse correlation of both age at onset of motor symptoms and age at death with the *HTT* CAG repeat length in HD subjects [1], [2], [3], [4], [5], [6]. This continuous correlative relationship strongly supports a role for dominant CAG length-dependent mechanisms in determining the rate of disease processes that result in manifestation of neurological symptoms. In addition, the continuous relationships between *HTT* CAG repeat lengths and molecular energy phenotypes also extend across the range of expanded disease alleles and into the normal *HTT* CAG repeat range (CAG<36), in a panel of blood-derived lymphoblastoid cell lines [7]. These suggested that the *HTT* CAG repeat is a functional polymorphism directly associated with an alteration of huntingtin function that leads eventually to disease phenotypes. These and other genotype-related phenotypes are also observed in induced pluripotent stem cell derived neuronal cell lines [8]. Thus, identifying biological processes that are influenced by the *HTT* CAG repeat in a length-dependent manner will significantly inform the molecular mechanisms underlying HD, and thereby, facilitate the development of effective treatments.

As a part of our ongoing efforts to comprehensively optimize the discovery of *HTT* CAG repeat length-dependent molecular changes, we undertook a global and unbiased approach to identify genes whose expression levels were continuously correlated with *HTT* CAG repeat lengths. We chose to study a panel of 107 human lymphoblastoid cell lines derived from HD subjects and normal controls, as there are currently many more such lines available than any other standardized cell types, making it possible to include samples to achieve a broad and continuous spectrum of CAG lengths. Although the effects of the *HTT* CAG repeat on gene expression in lymphoblastoid cells were relatively modest, a significant amount of variance in gene expression was attributable to the *HTT* CAG repeat length [9]. These findings indicated that: 1) *HTT* CAG repeat length-correlated gene expression changes exist, and 2) continuous analysis was able to identify *HTT* CAG repeat length-dependent molecular signals from other factors contributing noise, including genetic heterogeneity of the study subjects, thereby demonstrating the power of continuous analytical strategies. In addition, continuous analysis was able to detect modest but significant correlations between *HTT* CAG repeat lengths and genes that were not significant in a dichotomous analytical comparison, supporting the sensitivity of continuous analysis approaches. Taken together, these results demonstrated the power of continuous analysis strategies to capture *HTT* CAG length-correlated gene-expression signatures that conform to the criteria expected for effects of the mechanism that contribute to the HD disease process.

Despite their relevance to HD, continuous analysis approaches are not widely used in the HD field. Instead, dichotomous analysis methods comparing HD to normal controls are more widespread. We hypothesize that dichotomous analysis methods are not optimal for investigating HD where *HTT* CAG repeat length-correlated alterations are strongly implicated in playing an important role in HD pathogenesis. Thus, we performed a continuous analysis and a dichotomous analysis on the same data set comprising microarray gene expression data of 107 human lymphoblastoid cell lines derived from HD and normal controls. We then compared the results to evaluate the sensitivity and specificity of the dichotomous analysis method, relative to the continuous method based upon CAG length. In addition, based upon those results, we created a searchable internet website, HD CAGnome, where users can evaluate the relationships between genes of interest and *HTT* CAG repeat length in both continuous and dichotomous contexts.

## Materials and Methods

### Gene Expression Dataset

Gene expression profile data were generated from 107 Epstein–Barr virus-transformed human lymphoblastoid cell lines, which were maintained internally at the Massachusetts General Hospital. These cell lines were derived from 105 independent subjects, comprising 41 normal control samples (CAG<36) and 66 HD samples (CAG>35) [9]. The mean sizes of the longer and shorter alleles were 40.9 and 17.6 CAGs, respectively. The range of the longer alleles was 15–92. Cells were grown in RPMI-1640 media supplemented with 10% fetal bovine serum, 100 I.U./ml Penicillin, and 100 I.U./ml Streptomycin at 37°C. The *HTT* CAG repeat allele lengths for each sample were determined by a polymerase chain reaction assay, against sequenced allele size standards, as described previously [10]. For all analyses, only the longer CAG allele was utilized, as the shorter allele has been demonstrated not to contribute to HD age at onset [4]. For microarray gene expression profiling experiments, total RNA was extracted using TRIzol reagent (Invitrogen) and further purified using a RNeasy kit (Qiagen). All RNA samples passed quality control by 260/280 ratio (NanoDrop) and 28S/18S ratio (Agilent Bioanalyzer assay). Total RNA (5 µg) was converted into cDNA using SuperScript II reverse transcriptase (Invitrogen) and labeled probe was synthesized by Single-Round RNA Amplification and Biotin Labeling System (Enzo). Labeled probe (25 µg) was hybridized to Affymetrix U133+2 arrays as recommended by the manufacturer. All microarray data were processed together for background correction and normalization using gcRMA (R, 2.11.1; gcrma, 2.20.0) followed by batch effect correction [11].

### Statistical Analysis

We analyzed microarray expression data using two different approaches. For the continuous analysis approach, we performed 1) a Pearson’s correlation test and 2) a Spearman’s rank correlation test to evaluate the strength and significance of the continuous relationship between CAG repeat length and expression level of a given gene. Based on dominance in HD, we primarily used the length of longer CAG repeat in a given subject to calculate the strength of correlation with gene expression levels. In addition, due to the fact that the longer CAG repeat is highly correlated with the sum of two CAGs in our samples, we observed a high degree of similarity between two sets of correlation coefficients based on 1) the longer CAG or 2) the sum of CAGs. Therefore, we presented the results using the longer CAG repeat. The continuous analysis results are presented in a scatter plot and summary statistics of correlation tests in the HD CAGnome website. For the dichotomous analysis approach, we performed a Student’s t-test, comparing HD (66 samples; CAG>35) to normal controls (41 samples, CAG<36) to determine whether the expression level of a gene in HD differs significantly from that in normal controls. Similarly, a box plot and a summary statistics table are presented in the HD CAGnome website. All computational and statistical analyses were performed using R (version 2.11.1).

### Comparison of Continuous Analysis Results to those of Dichotomous Analysis

In order to estimate from dichotomous analysis 1) what percentage of CAG-length correlated probes were detected and 2) what percentage of probes predicted as significant were in fact not CAG-length correlated, we randomly selected variable numbers of HD and normal samples from our dataset and calculated the significance level of each probe using Student’s t-test. These procedures were repeated 1,000 times for each sample size analysis. We varied the sample size from 3 to 41; for example, the first set of comparisons compared 3 randomly selected HD samples to 3 randomly selected normal samples (n = 3), the second set of comparisons compared 4 randomly selected HD samples to 4 randomly selected normal samples (n = 4), and so on. Since our dataset had 41 normal control samples but 66 HD samples, we compared up to 41 HD and 41 normal samples (n = 41). In each iteration, p-values of nominally significant probes were recorded and compared to p-values of the same probes obtained from continuous analysis by CAG length of all samples using the Pearson’s correlation test. We calculated 1) the fraction of all probes significant in the continuous analysis that were also significant by dichotomous analysis (i.e., continuous dichotomous shared), 2) the fraction of all probes significant in the continuous analysis that were not significant by dichotomous analysis (i.e., continuous specific), and 3) the fraction of all probes significant in the dichotomous analysis that were not significant in the continuous analysis (i.e., dichotomous specific). Based on these results, statistical models were constructed in order to understand relationships between parameters and to predict the sample sizes that allow dichotomous analysis to detect 80% of the positives from continuous analysis and to reduce the rate of predicting differences that are not significant in the continuous analysis to 20%. Although relationships may not be linear, linear models were used because parametric models provide convenient tools for extrapolation, and linear models generated model fits with high R-squared values.

### Website Construction

For each gene, the gene name, official HUGO symbol, and Affymetrix probe set ID are dynamically generated via Perl CGI scripts and presented. Pre-computed summary statistics and plots are presented upon user’s input and selection. All raw data and normalized data are available at Gene Expression Omnibus (GSE34721). In addition, normalized data with summary statistics are available for download from HD CAGnome.

## Results

**Figure 1:**
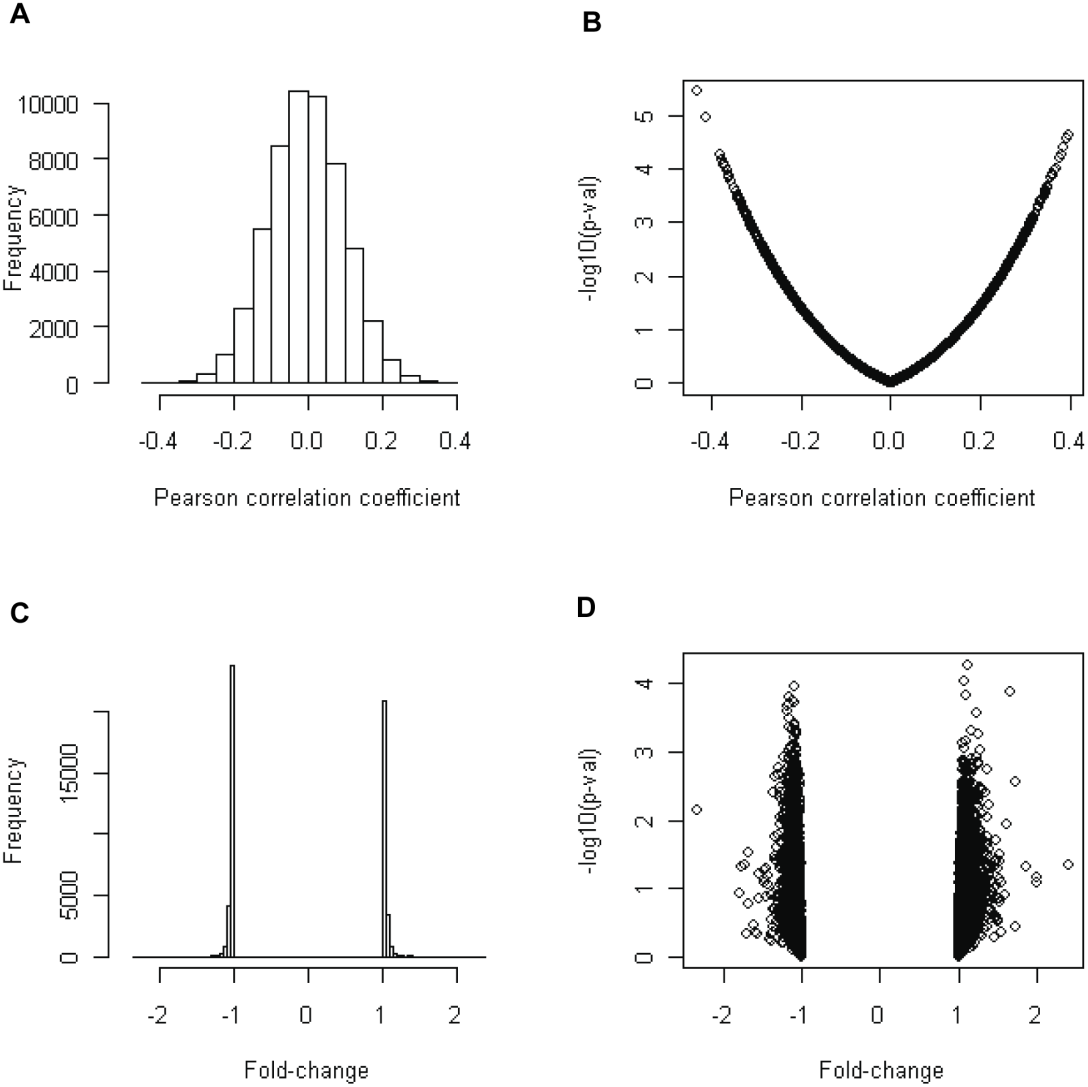
Overall characteristics of continuous and dichotomous analysis results. Pearson’s correlation test was performed for continuous analysis. The distribution of correlation coefficient (A) and correlation coefficient vs. significance (i.e., −log10(p-value)) (B) were plotted. For dichotomous analysis, Student’s-t test was used, and the distribution of fold-changes (C) and fold-change vs. significance (−log10(p-value)) (D) were plotted.

**Figure 2:**
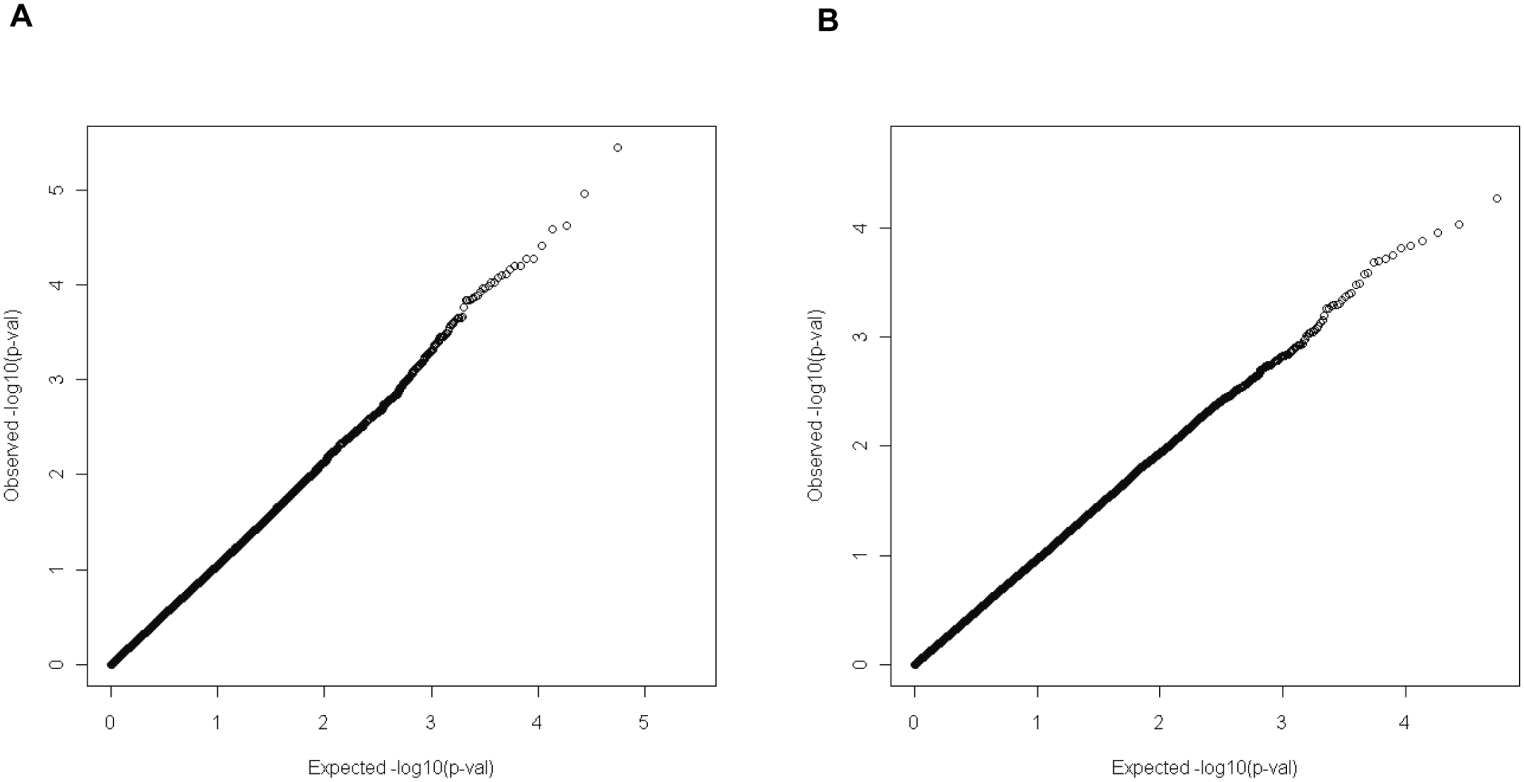
Quantile-quantile plot. Expected p-values assuming a uniform distribution (X-axis; expected −log10(p-value)) were compared to observed p-values (Y-axis; observed −log10(p-value)) in the continuous analysis (A) and dichotomous analysis (B) to evaluate the levels of inflation in test statistics. Expected p-values were calculated based on the uniform distribution assuming each test was independent of others.

**Figure 3:**
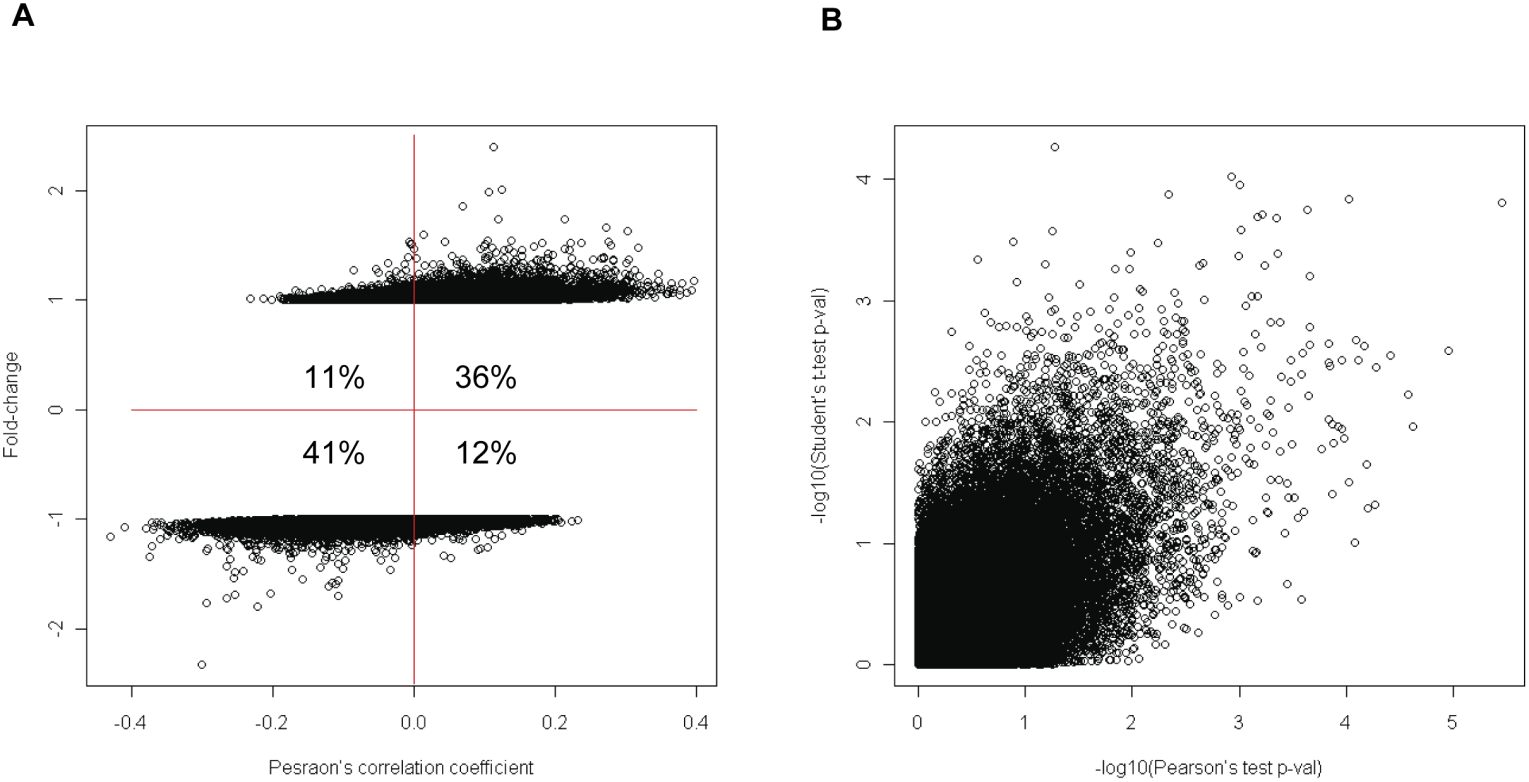
Comparison of test statistics. (A) Pearson’s correlation coefficients in the continuous analysis were compared to fold-changes in the dichotomous analysis to evaluate overall similarity in direction of changes between two analytical methods. The number in each quadrant represents the percentage of probes relative to all probes analyzed. (B) Significance levels in the continuous analysis (−log10(Pearson’s test p-value); X-axis) were compared to those in the dichotomous analysis (−log10(Student’s t-test p-value); Y-axis) in order to evaluate overall similarity in significance between two analytical methods.

We performed continuous analysis and dichotomous analysis, respectively, to identify genes whose expression levels were correlated with *HTT* CAG repeat length (Pearson’s correlation test) or whose expression levels were significantly different between HD (CAG>35) and controls (CAG<36) (Student’s t-test). Figure 1 summarizes characteristics of overall test statistics from continuous analysis (Fig. 1A and 1B) and dichotomous analysis (Fig. 1C and 1D). The range of Pearson’s correlation coefficients in the continuous analysis was −0.431 to 0.396 (Fig. 1A), and the p-value of the most significantly correlated probe was 0.00000358 (Fig. 1B). In the dichotomous analysis, the range of fold-change was −2.33 to 2.39 (Fig. 1C), and the most significant p-value was 0.0000536 (Fig. 1D). Comparison of observed p-values to expected p-values based on the uniform distribution revealed that the overall test statistics were not inflated in either the continuous analysis (Fig. 2A) or the dichotomous analysis (Fig. 2B). Comparison of Pearson’s correlation coefficients in the continuous analysis to fold-changes in the dichotomous analysis showed that 77% of microarray probes showed the same direction of change (Fig. 3A; top-right and bottom-left quadrants), and 23% of microarray probes showed an opposite direction of change (Fig. 3A; top-left and bottom-right quadrants). In addition, significance levels for probes in the continuous analysis showed a modest correlation with those in the dichotomous analysis (Fig. 3B), suggesting overall similarity between the continuous analysis results and the dichotomous analysis results.

**Figure 4:**
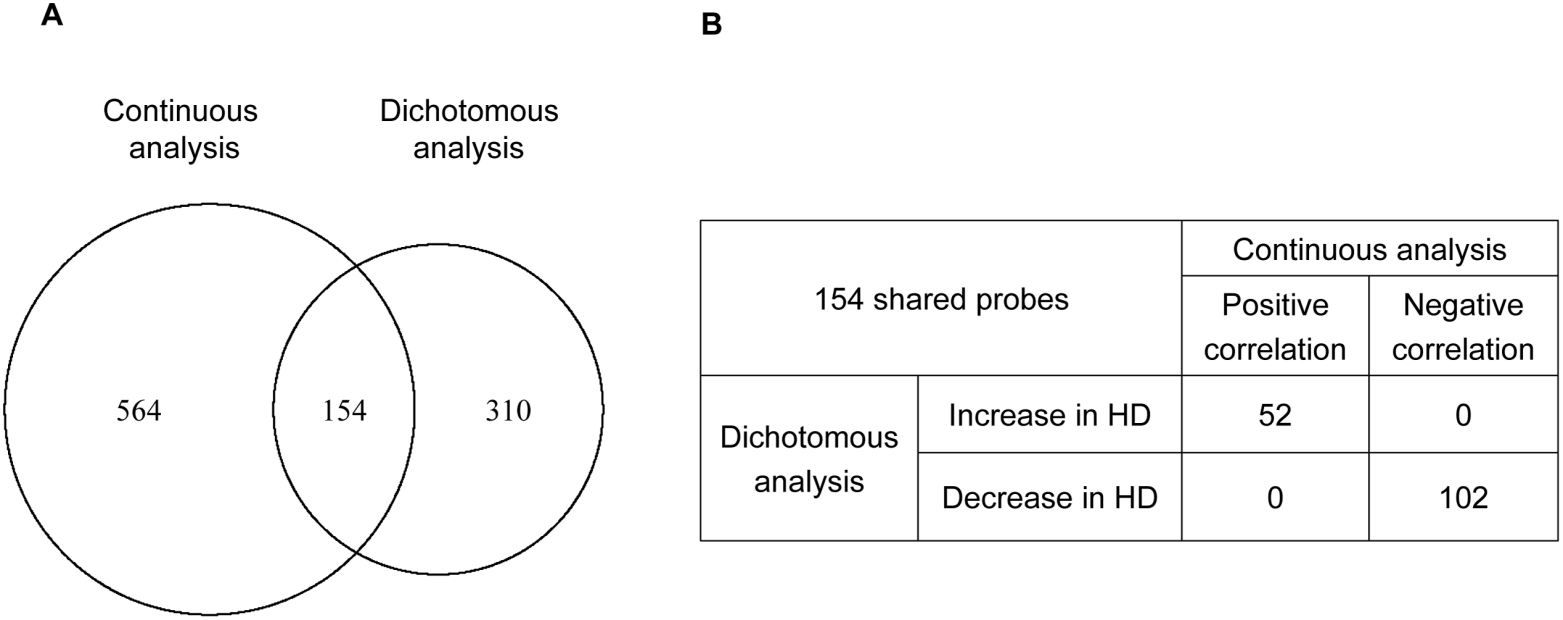
Significant probes in continuous and dichotomous analysis. Pearson’s correlation test and Student’s-t test were used for continuous and dichotomous analysis, respectively. (A) From left to right, numbers in Venn diagram indicate the number of probes significant only in continuous analysis, probes significant in both continuous and dichotomous analysis, and probes significant only in dichotomous analysis by p-value cut-off of 0.01. We did not observe differences between shared genes and specific genes in terms of p-values, correlation coefficients, and fold-changes. (B) Probes significant in both analyses (154 probes) were further categorized based on the correlation coefficient and fold-change.

Next, we compared significant probes. Nominal p-value of 0.01 was used to identify significant genes in each analysis. 718 microarray probes were significantly correlated with *HTT* CAG repeat length in the continuous analysis (315 probes with positive correlation and 403 probes with negative correlation). 464 probes were significant in the dichotomous analysis (175 increase in HD and 289 decrease in HD). Between significant probes in continuous analysis and those in dichotomous analysis, 154 probes were shared as shown in Fig. 4A. Probes significant in both analyses showed consistent direction of change (Fig. 4B). For example, 52 probes that showed nominally significant positive correlation with *HTT* CAG repeat length were significantly increased in HD compared to normal controls; 102 probes that were negatively correlated with *HTT* CAG repeat length also showed decreased expression levels in HD (Fig. 4B). Considering that truly correlated genes must necessarily show differences in dichotomous analysis, these observations indicate that statistics from correlation analysis and dichotomous analysis for commonly significant genes were not significantly affected by a subset of influential data points.

Next, we evaluated potential limitations of the dichotomous analysis strategy in HD where the importance of continuous changes is strongly supported by human genotype-phenotype relationships. We calculated two metrics of dichotomous analysis: 1) the percentage of the probes significant in continuous CAG-length analysis that were also significant in dichotomous analysis, 21.4% (and by subtraction, the percentage not detected) and 2) the percentage of probes that were significant in the dichotomous analysis but not in the continuous analysis, 66.8%. Thus, the dichotomous analysis failed to identify 78.6% of the significant differences detected by continuous analysis, and only 33.2% of the genes detected by dichotomous analysis actually showed significant correlation with CAG length.

**Figure 5:**
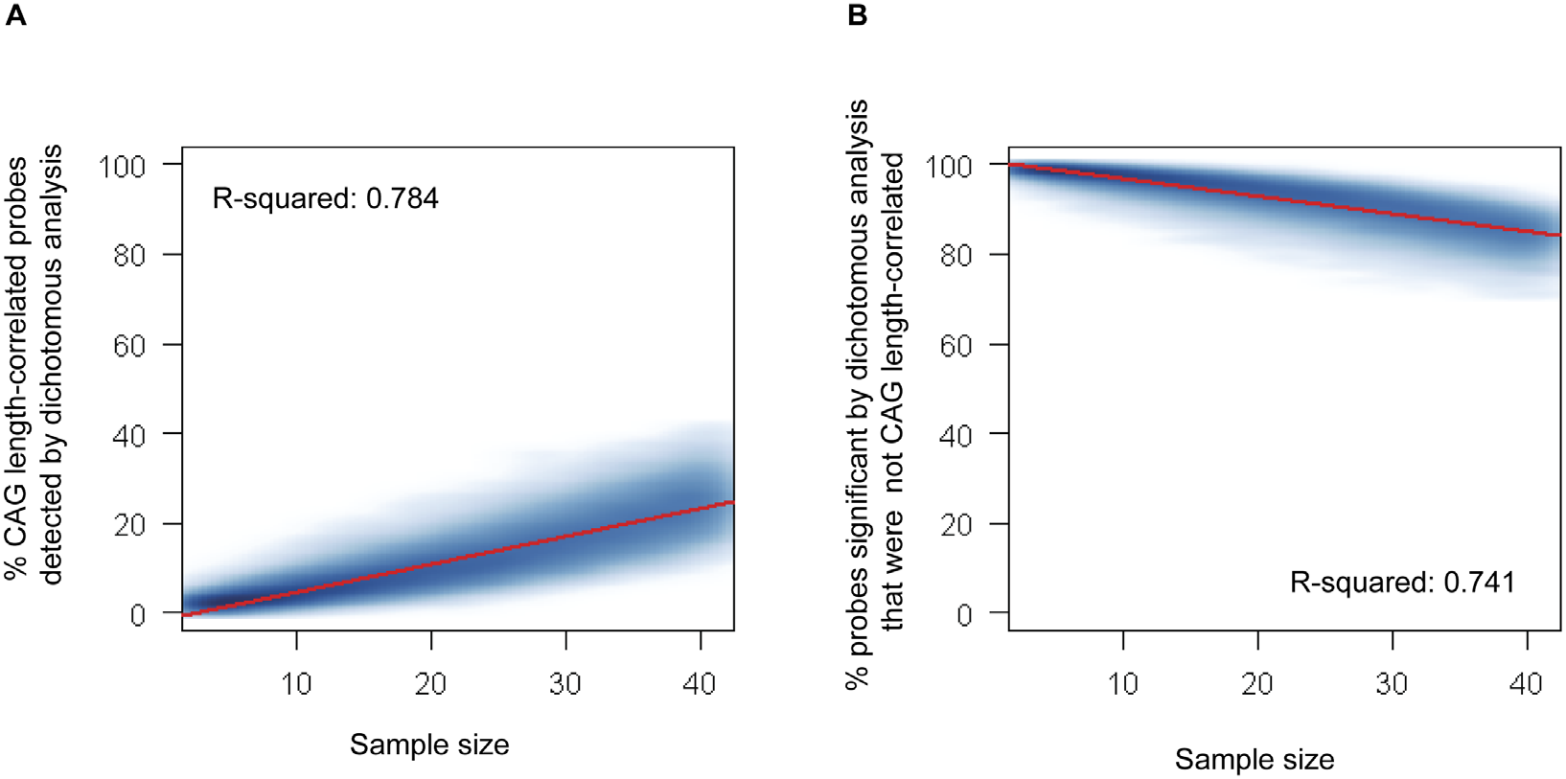
Efficiency of dichotomous analysis in capturing significantly correlated genes. From 107 samples, we randomly selected equal numbers (n = 3 to 41) of HD and controls to perform dichotomous analysis, repeating 1,000 times for each sample size, and compared to continuous CAG length analysis, as described in the text and methods, plotting the resulting percentages in scatter density plots. (A) Variation in the percentage of CAG-length correlated significant differences from continuous analysis that are detected by dichotomous analysis vs. sample size. (B) Variation in the percentage of differences judged significant by dichotomous analysis that are not CAG-length correlated by continuous analysis vs. sample size. Red lines represent linear regression models describing the relationship between sample size and the corresponding performance metric.

We predicted that dichotomous analysis may show even poorer performance when the sample size is small. To test this prediction, we performed simulation experiments employing dichotomous analyses on variable numbers of randomly selected samples, and determined the effect of sample size on the above metrics. For each random sampling, we performed dichotomous analysis utilizing Student’s t-test and calculated the metrics by comparing to the results of all sample correlation analysis. For each sample size, starting with n = 3 (i.e., 3 HD vs. 3 controls) and ending with n = 41 (i.e., 41 HD vs. 41 controls), the dichotomous analysis and comparison to the all sample continuous analysis were repeated 1,000 times. Figure 5 shows variation in the two metrics with sample size, where the frequency of the percentages is represented as smoothed color density in which darker color represents higher frequency. For a given sample size, the outcomes were quite variable, but there was a clear trend for increased sample size in the dichotomous analysis to result in detection of a higher percentage of the significant differences from continuous analysis. Similarly, increasing sample size in the dichotomous analysis also resulted in a decrease in the percentage of genes seen to be significant in the dichotomous analysis but not the continuous analysis. Since we were interested in determining efficiency of dichotomous analysis approaches in capturing the continuous effects of *HTT* CAG repeat lengths on gene expression, we constructed regression models based on the simulation analysis (Fig. 5; red lines). Then we estimated the number of samples that would be required to achieve two arbitrarily selected performance criteria for the dichotomous analysis: 1) identification of 80% of the significant differences detected in continuous analysis and, 2) a yield of only 20% of differences predicted as significant that are actually non-significant by continuous analysis. By extrapolating models, we estimated that approximately 133 and 206 samples in each group would be required to achieve these respective limits, indicating that analysis in a dichotomous fashion requires significantly more samples than continuous analysis to capture the CAG-length correlated effects of the *HTT* repeat on gene expression (Fig. 5). Taken together, these results reveal potential limitations of dichotomous analytical approaches in studying the effects of a mutation/polymorphism with a continuum of lengths, reinforcing the importance of the use of appropriate analytical approaches in HD and potentially in other of the growing number of triplet repeat expansion disorders.

**Figure 6:**
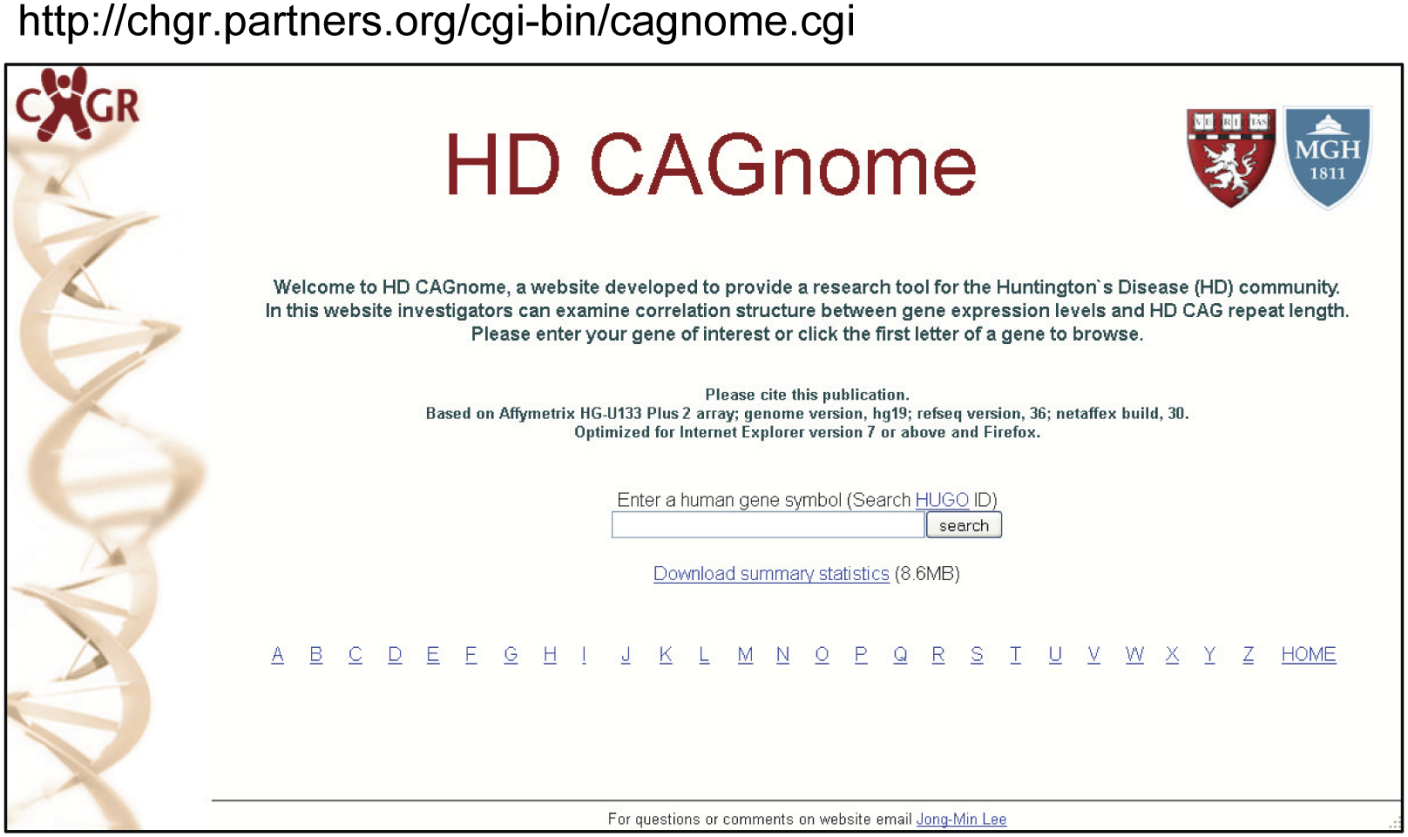
HD CAGnome. HD CAGnome is accessible at http://chgr.partners.org/cgi-bin/cagnome.cgi.

Finally, we created a searchable website (HD CAGnome) to encourage the application of continuous analysis approaches in HD by presenting correlation analysis results from our microarray gene expression data analysis. The web interface (Fig. 6) supports two options: 1) entering a human gene symbol in the search window or 2) browsing a list of genes. Selecting a gene using the search window and browse function retrieves identical information. The link to the HUGO Gene Nomenclature Committee (HGNC) is provided as a resource for obtaining the symbol of a gene of interest. The search results in HD CAGnome will show the full gene names, official gene symbols, Entrez IDs, and Affymetrix probe IDs. Primarily, HD CAGnome was designed to provide information concerning the correlation structure between gene expression levels and *HTT* CAG repeat length. The results (scatter plot) of the summary statistics from continuous analyses (Pearson’s correlation analysis and Spearman’s correlation analysis) are presented in the left side of the window. A box plot with summary statistics of dichotomous analysis (Student’s t-test) is also presented in the right side of window. HD CAGnome is freely available for online searching and downloading at http://chgr.partners.org/cagnome.cgi.

## Discussion

The strong negative correlation between age at onset of HD motor symptoms and *HTT* CAG repeat size supports a role for CAG length-dependent continuous mechanisms in determining the rate of the underlying disease process in HD. However, dichotomous analysis comparing HD versus normal controls is a standard in the field, where differences in the *HTT* CAG repeat length between expanded alleles are rarely taken into account [12], [13], [14], [15], [16], [17], [18]. Based upon the knowledge that the rate-determining processes in pathogenesis leading to diagnostic onset are dependent on the *HTT* CAG repeat length, we predicted that dichotomous analytical methods have a lower sensitivity in identifying important biological changes that are relevant to HD compared to continuous analytical methods. To test this prediction, we compared a set of *HTT* CAG repeat length-correlated genes to differentially expressed genes, and observed a minimal overlap between nominally significant genes in continuous and dichotomous analysis. As the *HTT* CAG repeat length-correlated genes fulfill the genetic criteria for being associated with the pathogenic process in HD, these findings suggested that dichotomous analysis likely generates results with significant false negatives and false positives that could confound further investigation. In addition, sample size directly influenced the levels of such false negative and false positive metrics in the dichotomous analysis, i.e., an increase of sample size by 10 (10 HD and 10 controls) had the effect of decreasing the corresponding metrics by 6% and 4%, respectively. These observations imply that dichotomous analysis of HD will reveal *HTT* CAG repeat length-dependent changes only with significantly larger sample sizes.

Gene expression can be influenced by many factors, both genetic and environmental. We recently showed using a continuous analysis of microarray gene expression data that ∼20% of variance in gene expression could be attributed to *HTT* CAG repeat length [9]. Together with the relative performance of dichotomous analysis in these experiments, the modest size of this CAG length effect suggests that most of the differences significant by dichotomous analysis are ‘noise’. Pathway analysis, gene set enrichment analysis, and network analysis are popular in the field because the power and sensitivity would be increased if properly performed based on correct and unbiased gene level analysis results. However, we predict that such limitations of dichotomous analytical methods may result in elevated inaccuracy when genome-wide scale large data are analyzed by this approach since the inherent high rate of false positives would be compounded in subsequent pathway and molecular network analyses, reducing their reliability. Understanding how cells modulate the expression of genes in response to the expanded *HTT* CAG repeats is important as this can provide insights into underlying mechanisms and pathways of HD. In addition, genes with altered levels of expression are of great utility as HD biomarkers. As we described previously, the *HTT* CAG repeat length has a modest impact on the gene expression [9], indicating that analysis with insufficient power may generate significant numbers of false negatives. In addition, highly heterogeneous nature of human subjects poses a challenge to the discovery of genes whose expression levels are altered in blood and brains of HD subjects [12], [13], [17]. In this context, the use of CAG repeat length as a continuous variable may reduce false discoveries because influences of confounding factors on statistical models would be reduced in a continuous analysis.

In summary, our analysis reveals that there are potential limitations in using dichotomous analysis approaches when studying HD, especially when the number of samples may be constrained by the availability of a given tissue or cell type. Knowledge from direct comparisons between continuous and dichotomous analysis results is important as this will provide guidelines for molecular studies aiming at investigating the *HTT* CAG repeat length-dependent changes, for example in choosing samples that represent a full-spectrum of CAG repeat lengths. Motivated by these findings, we created a website, namely ‘HD CAGnome’ that can be used to examine the strength and direction of correlation between *HTT* CAG repeat length and expression levels of a given gene. We also provide a summary of dichotomous analysis results so users can compare two results. We believe that this novel resource will provide convenient ways of evaluating correlation structure of candidate genes and generating new hypotheses, that may be tested in other cell types, including neuronal cells, and tissue samples, for example studies with postmortem brain tissue. Therefore, our findings presented in HD CAGnome will facilitate the use of analytical approaches that are relevant to HD and contribute to identifying genes that can be used for therapeutic treatments and/or biomarkers.
